# Virtual reality-based training for radiopharmaceutical administration: development and educational effectiveness

**DOI:** 10.1371/journal.pone.0321101

**Published:** 2025-03-31

**Authors:** Akihiro Kakimoto, Daisuke Fujise, Shin Hasegawa, Yasuo Okuda, Yuto Ohta, Rikuha Tani, Miyu Niwase, Kazutoshi Miyamoto, Ryota Konishi, Masao Funahashi

**Affiliations:** 1 Department of Radiological Sciences, Faculty of Medical Science Technology, Morinomiya University of Medical Science, Osaka, Japan; 2 Department of Biofunctional Imaging, Preeminent Medical Photonics Education and Research Center, Hamamatsu University School of Medicine, Hamamatsu, Japan; 3 IT Planning Section, Department of Information Technology and Management, National Institutes for Quantum Science and Technology, Chiba, Japan; Gachon University, KOREA, REPUBLIC OF

## Abstract

In Japan, task shifting and sharing are promoted to reduce the workload of physicians. Radiological technologists have been assigned new responsibilities, such as securing venous access for radiopharmaceutical administration. This study aimed to develop a prototype Virtual Reality (VR) training system that allows safe and repeatable training for radiological technologists. Additionally, the educational effectiveness of VR training was evaluated, and the concentration levels of the participants were assessed using multiple physiological indicators. Overall, 17 male and 12 female participants (mean age 20.1 ±  0.9 years) were enrolled in this study and classified into two groups—video-based and immersive VR system groups—both of which simulated radiopharmaceutical administration. Concentration and tension levels were evaluated using electroencephalography (EEG) data, salivary amylase levels, and mood assessments. The educational effectiveness was evaluated using a multiple-choice cognitive test. Compared with the resting levels, the alpha/beta ratio of EEG (indicating relaxed concentration) was significantly decreased by 19% in the video-based VR and increased by 40% in the immersive VR groups (both p < 0.05). No significant difference was observed in salivary amylase levels between the two groups. The cognitive test scores, increased by 2.0 and 3.4 points in the video-based VR and immersive VR groups, respectively; a significant difference was observed between both groups (p <  0.05). However, no correlation was found between the EEG ratio and test performance. Thus, immersive VR promotes a more relaxed and concentrated state and was found to have higher educational effectiveness than video-based VR. This suggests that participatory VR training may be more effective than observational VR training. Further research should explore the relationship between educational effectiveness and the evaluation of medical skills.

## Introduction

In Japan, the Medical Care Act and related laws have been revised to promote the establishment of a system that efficiently provides high-quality and appropriate medical care [1]. Consequently, task shifting and sharing between physicians and other healthcare professionals have become more prevalent. The revision of the Radiological Technologists Act (effective October 1, 2021) introduced new responsibilities for radiological technologists, including securing venous access for radiopharmaceutical administration and performing needle removal and hemostasis.

However, a survey conducted across 10 facilities in the United States covering approximately 30,000 cases revealed that protocols and training methods for these tasks have not yet been established [2]. The necessity of controlled areas for non-sealed radioactive isotope handling, as well as the risks associated with puncturing and radiation exposure, pose high costs and ethical challenges. Against this backdrop, radiology technologist training schools in Japan are currently facing a situation in which practical training in these medical techniques is required following the introduction of new training programs and curricula.

We focused on Virtual Reality (VR) technology, which provides safe and repeatable training. Numerous VR training systems related to radiation have been reported, and it has been proposed that radiation visualization (normally invisible) enhances awareness of radiation exposure [3–7]. However, there is limited scientific evidence on the effectiveness of immersive training, such as on tension and concentration, and its correlation with educational effectiveness during VR operation [8].

In this study, we developed two types of training materials: a 360° video-based VR system that allows radiological technologists to trace tasks from their perspective, and an immersive VR system that provides a near-realistic experience using a VR-specific glove. This study aimed to investigate the impact of immersion on educational effectiveness, representing a novel immersion-dependent approach to scientifically confirm learning outcomes to facilitate safe and efficient training in radiopharmaceutical administration tasks in radiological technologist training schools, thereby enhancing students’ education.

## Materials and methods

### Ethics statement

This study was reviewed and approved by the University Ethics Committee [2022-059] to which the principal investigator is affiliated. The recruitment period for this study spanned from December 1, 2023, to July 18, 2024. Research participants were recruited through an open call within the university, and informed written consent was obtained from the participating students after a thorough explanation of the study and its lack of impact on their academic performance. As all the participants were aged 18 years or older, parental consent was not required. Although no physical intrusion such as pain or radiation exposure was experienced through the VR immersive system, the possibility of VR-induced motion sickness was acknowledged. Therefore, a support system involving medical professionals was established to ensure the safety of participants during data collection.

### Development of an immersive VR system

**Fig 1:**
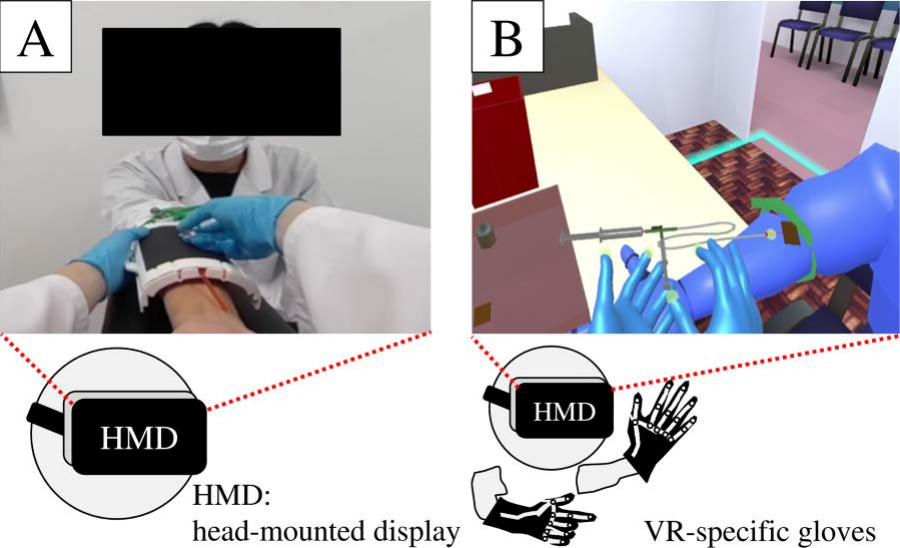
VR training system for radiopharmaceutical administration. (A) A 360° video-based VR system that allows for task tracing from the perspective of a radiological technologist. (B) Immersive VR system that allows users to simulate operations.

The VR environment of the immersive VR system was constructed using a scenario identical to that of the 360° video-based VR system, and we employed the following device: a PC running Windows Pro 10 64-bit equipped with a GeForce RTX2080 (NVIDIA) graphics card. The VR hardware included a head-mounted display VIVE Pro Eye (HTC) for the VR goggles, Molison Han s Vib + [L] (Feel the same) for VR-specific gloves and VIVE Tracker 3.0 (HTC) for glove tracking. The platform used for development was Unity Version 2023.3.7f1 (Unity Technology), and the software development kits (SDKs) utilized were SteamVR Plugin 2.7.4 (sdk1.14.15), SteamVR 2.2.3, and Mollisen SDK. Buttons with comments detailing the tasks per scene were placed in the VR environment. Pressing these buttons advanced the scenario by creating an event-driven structure. For instance, buttons such as “alcohol disinfection,” “radiopharmaceutical administration,” and “saline flush” are provided. By pressing these buttons, the users were trained on these tasks in a step-by-step manner, with each procedure simulated as part of the training. Fig 1B shows a portion of the screen viewed by the operator of the immersive VR system.

### Experimental protocol

Thirty-two students attending a radiological technologist training school (17 males, 15 females), with a mean age of 20.0 ±  1.0 years, were enrolled in this study (S1 Table). The participants were divided into two groups: 360° video-based and immersive VR systems. To observe the fluctuations in stress and concentration pre- and post-VR operation, this study employed physiological indicators, such as salivary amylase [9–10] and electroencephalography (EEG) [11–12], and to assess psychological states, it employed the profile of mood states in the second edition (POMS2) [13–17] and two-dimensional mood scale–short-term (TDMS-ST) [18]. Additionally, to evaluate the educational effectiveness of the pre- and post-VR operations, six multiple-choice questions with four options were prepared. For each question, the participants were asked to respond with one of the following: Remember, Know, or None (do not remember at all) [19–20].

**Fig 2:**
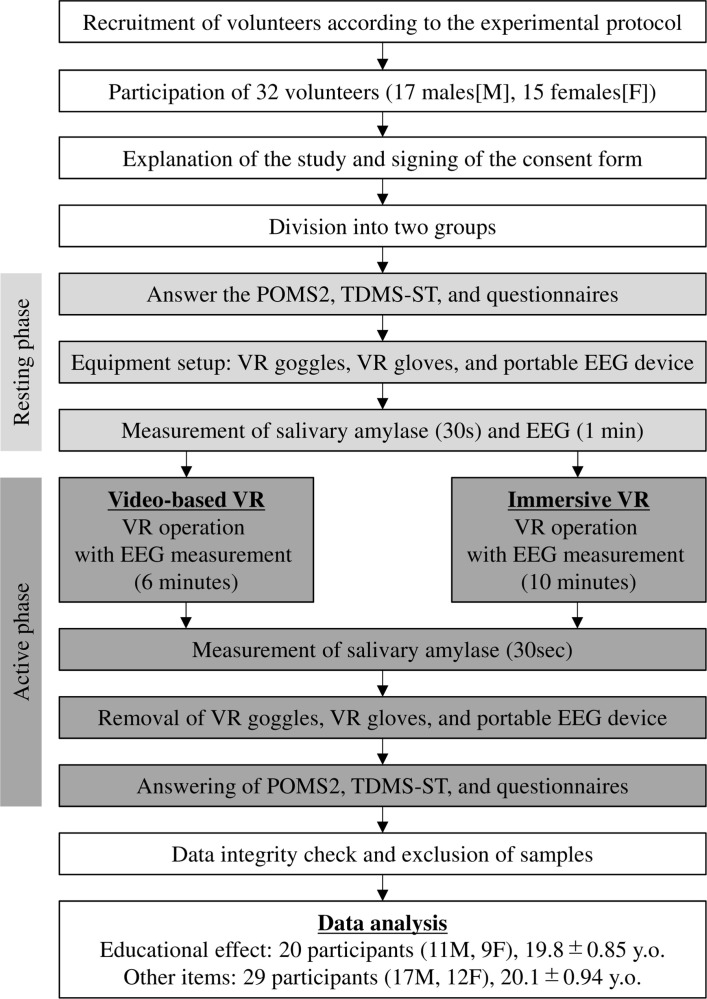
Experimental protocol.

Fig 2 shows a flowchart of the experimental protocol. Initially, to assess baseline tension and concentration levels, participants completed the POMS2, TDMS-ST, and educational effectiveness evaluation questionnaires. Subsequently, participants were fitted with a VR headset, VR-specific gloves, and an EEG device (Brain, FocusCalm). Subsequently, saliva was collected on a specialized chip for 30 s to measure the salivary amylase concentration using a salivary amylase analyzer (NIPRO, 59-010). In addition, EEG measurements in the resting state were recorded for 1 min using a FocusCalm EEG device. The data obtained during this phase were considered the baseline pre-VR resting state values.

The EEG was measured during the VR operation. Furthermore, the video-based VR system involved a 6-min viewing session, whereas the immersive VR system had an approximately 10-min experience period. Immediately post-VR operation, saliva was collected for analysis. After removing the VR equipment, the participants completed the POMS2, TDMS-ST, and educational effectiveness evaluation questionnaires. Finally, data integrity was checked and participants were excluded if their EEG data were missing, salivary amylase levels were below the detection limit, or errors were found in their questionnaire responses. Ultimately, data from 20 participants (11 males, 9 females, mean age 19.8 ±  0.85 years) were analyzed for educational effectiveness, while data from 29 participants (17 males, 12 females, mean age 20.1 ±  0.94 years) were analyzed for other measurement items. None of the participants in this study had prior experience wearing head-mounted displays (HMDs), VR-specific gloves, or immersive VR applications. The video-based VR session lasted 4 min, whereas the immersive VR session lasted approximately 6 min.

Flowchart illustrating the experimental procedure and comparison of salivary amylase, POMS2, TDMS-ST, multiple-choice questionnaires, and EEG results between the resting state and during/post-VR operation.

### Data analysis

#### Salivary amylase.

The difference in salivary amylase levels between the resting and active phases was calculated and compared between the groups. For significance testing, a t-test was used with a significance level of p <  0.05.

#### Psychological state.

For POMS2, the differences between the resting and active phases were calculated for the following seven evaluation items: anger-hostility, confusion-bewilderment, depression-dejection, fatigue-inertia, tension-anxiety, vigor-activity, and friendliness. Additionally, the differences between the resting and active phases were calculated for the four evaluation items of the TDMS-ST (vitality, stability, pleasure, and arousal levels). A t-test was performed between the two groups per measure, and the results were corrected for multiple comparisons using the Bonferroni method.

#### EEG.

A simple EEG device was used to minimize interference from the VR headset. The electrode locations were positioned at Fp1, Fpz, and Fp2 according to the international 10-20 system, and the device collected noise-reduced alpha (α), beta (β), gamma, delta, and theta wave component ratios every 0.2 s. Generally, α waves are associated with relaxation and concentration, while β waves indicate tension and stress [21]. The study focused on the α/β ratio as an indicator of concentration and tension [22–23], calculating the difference between the resting and active phases as well as comparing the two groups.

#### Educational effectiveness.

Likert scales are commonly used in attitude surveys, satisfaction assessments [24], and educational effect questionnaires in the VR field [25]. To evaluate VR system performance and training effects, Likert scales typically include multiple-choice questions or open-ended questionnaire formats [26–28]. Six questions were developed, each containing four answers. The total scores pre- and post-VR operation were calculated, and the changes in these scores were compared between the two groups to assess educational effectiveness.

## Results

### Salivary amylase

**Fig 3:**
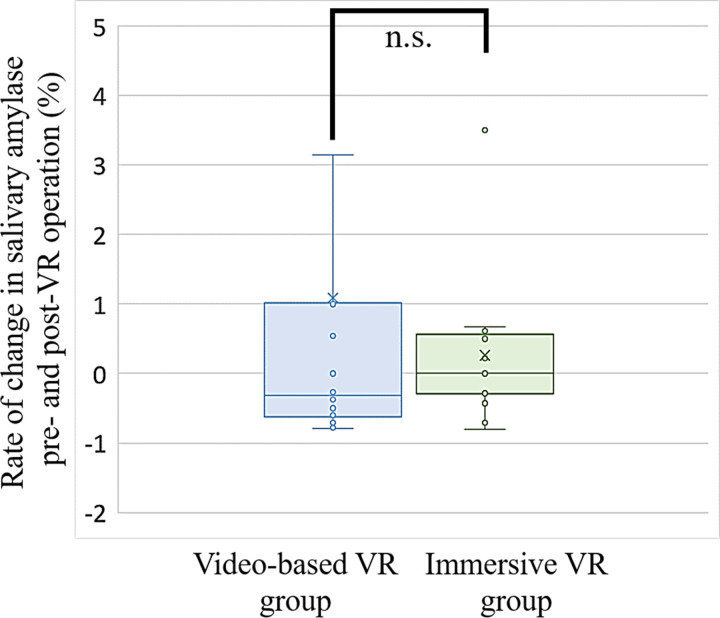
Rate of change in salivary amylase pre- and post-VR operation.

The average salivary amylase levels in the video-based VR group were 19.2 ±  16.0 KIU/L pre-VR session and 17.4 ±  14.5 KIU/L immediately after, showing no statistically significant difference, whereas in the immersive VR group, the average levels were 19.9 ±  21.6 KIU/L pre-VR session and 23.0 ±  28.1 KIU/L afterward. Although a slight increase was observed in the latter group, this difference was not statistically significant. The rates of change in salivary amylase levels pre- and post-VR, using the resting phase as the baseline, are presented for each data point in Fig 3 and S2 Table. Blue represents the video-based VR group and green represents the immersive VR group. Horizontal lines within boxes indicate first, median, and third quartiles. Data points exceeding 1.5 times the interquartile range are considered outliers, and the maximum and minimum values are depicted. The rate of change in the salivary amylase levels between the two groups was not statistically significant.

Box plot showing changes in salivary amylase levels relative to the baseline resting phase pre-VR. Blue: Video-based VR group; Green: Immersive VR group. Horizontal lines within boxes indicate first, median, and third quartiles. Exclusion of outliers from maximum and minimum values.

### Psychological state

Table 1 and S3 Table present the average POMS2 scores pre- and post-VR operation. The mood scale items included anger-hostility, confusion-bewilderment, depression-dejection, fatigue-inertia, tension-anxiety, vigor-activity, friendliness, and total mood disturbance. No significant differences were observed in any of these items either pre- or post-VR operation or between the video-based and immersive VR groups. Additionally, no significant differences were observed between the two groups in terms of individual score fluctuations for each mood scale item.

**Table 1:** Average scores of POMS2 pre- and post-VR operation.

Mood scale items	Average score of video-based VR group			Average score of immersive VR group		
	Resting phase	Active phase	Score changes	Resting phase	Active phase	Score changes
**Anger, hostility**	2.71 ± 2.92	1.86 ± 2.66	-0.86 ± 2.98	1.73 ± 2.40	1.40 ± 2.85	-0.33 ± 1.72
**Confusion, bewilderment**	7.43 ± 6.91	5.79 ± 5.19	-1.64 ± 2.53	3.21 ± 3.17	2.87 ± 3.88	-0.13 ± 2.13
**Depression, dejection**	6.86 ± 4.88	3.57 ± 4.89	-3.29 ± 4.66	2.00 ± 2.95	2.07 ± 3.26	0.07 ± 1.39
**Fatigue, inertia**	6.93 ± 5.77	6.36 ± 5.46	-0.57 ± 3.37	3.40 ± 3.56	3.00 ± 3.09	-0.40 ± 2.16
**Tension, anxiety**	7.64 ± 4.62	6.57 ± 5.20	-1.07 ± 2.67	4.40 ± 3.54	3.00 ± 3.21	-1.40 ± 3.24
**Vigor, activity**	7.50 ± 5.33	8.00 ± 5.11	0.50 ± 3.39	9.20 ± 4.26	8.00 ± 3.76	-1.20 ± 3.05
**Friendliness**	11.3 ± 4.58	12.2 ± 5.09	0.93 ± 2.59	11.3 ± 3.06	10.6 ± 3.91	-0.67 ± 1.72
**Total mood disturbance**	24.1 ± 23.4	16.1 ± 21.3	-7.92 ± 14.9	5.40 ± 14.3	4.33 ± 14.7	-1.07 ± 8.76

POMS2: Profile of mood states second edition. The mood scale items included anger-hostility, confusion-bewilderment, depression-dejection, fatigue-inertia, tension-anxiety, vigorous-activity, friendliness, and total mood disturbance.

Table 2 and S4 Table present the average TDMS-ST scores pre- and post-VR operations. Mood scale items included vitality, stability, pleasure, and arousal. In the multivariate analysis, no significant differences were observed in any of the items either pre- or post-VR operation or between the video-based and immersive VR groups. However, individual analyses revealed a tendency toward a reduction in scores for vitality, stability, and pleasure levels in the immersive VR group compared with the video-based VR group.

**Table 2:** Average scores of TDMS-ST pre- and post-VR operation.

Mood scale items	Average score of video-based VR group			Average score of immersive VR group		
**Resting phase**	**Active phase**	**Score changes**	**Resting phase**	**Active phase**	**Score changes**
**Vitality level**	0.29 ± 4.27	2.21 ± 3.04	1.93 ± 3.08	2.40 ± 3.54	2.00 ± 2.73	-0.40 ± 2.20
**Stability level**	5.71 ± 2.89	5.79 ± 2.78	0.07 ± 1.59	6.27 ± 2.28	4.73 ± 3.20	-1.53 ± 2.47
**Pleasure level**	6.00 ± 5.22	8.00 ± 4.59	2.00 ± 3.53	8.67 ± 4.76	6.73 ± 4.23	-1.93 ± 4.11
**Arousal level**	-5.40 ± 5.09	-3.60 ± 3.59	1.85 ± 3.39	-3.9 ± 3.58	-2.70 ± 4.17	1.13 ± 2.23

TDMS-ST: Two-dimensional mood scale-short term. Mood scale items: vitality, stability, pleasure, and arousal

### EEG

**Fig 4:**
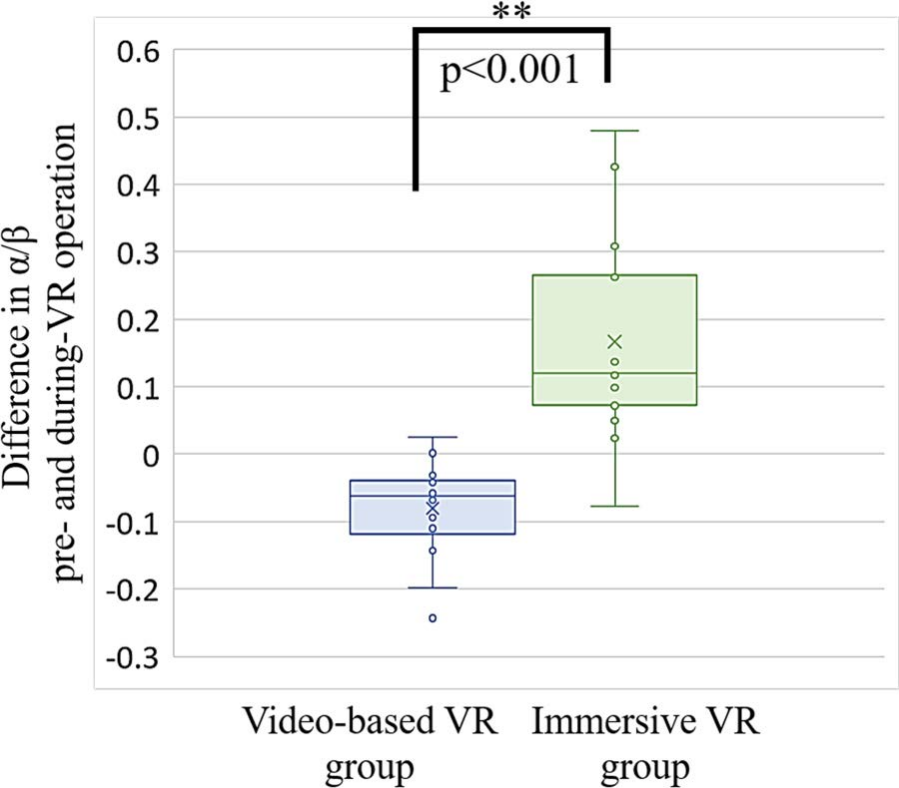
Difference in α/β pre- and during-VR operation.

The α/β ratios during the resting phase were 0.43 ±  0.17 in the video-based VR group and 0.42 ±  0.14 in the immersive VR group, with no significant difference observed between them. In contrast, during the active phase, the α/β ratio decreased by 19% to 0.35 ±  0.11 in the video-based VR group, whereas it increased by 40% to 0.59 ±  0.15 in the immersive VR group. A significant difference in the α/β ratio between the two groups was observed during the active phase (p <  0.001). Fig 4 and S5 Table present the individual data for the differences in α/β ratios between the active and resting phases, with the horizontal lines within boxes representing the first, median, and third quartiles. Data points exceeding 1.5 times the interquartile range are considered outliers, and the maximum and minimum values are shown. Furthermore, significant differences were observed in the changes in the α/β ratios between the active and resting phases of the two groups (p <  0.001).

Box plot showing the differences in α/β pre- and during-VR operation. Blue: Video-based VR group; Green: Immersive VR group. Horizontal lines within boxes indicate first, median, and third quartiles. Exclusion of outliers from maximum and minimum values.

### Educational effectiveness

Table 3 and S6 Table list the multiple-choice questions used to evaluate the educational effects and scores pre- and post-VR operations. The number of responses for each category (Remember/Know/None) is also listed. The questions were created by one radiological technologist (specializing in nuclear medicine) with prior experience with VR operations and three students from a diagnostic radiological technologist training school who were not included as participants. Overall, the questions were designed to assess knowledge that was not a well-known pre-VR operation and included content that was common to both groups. The questions ranged from simple, such as asking about the color of the tourniquet, to more medically specialized, such as about the locking operation of a three-way stopcock. The scores were calculated as 1 for correct answers and 0 for incorrect answers. The average increase in scores pre- and post-VR operation was 2.0 ±  1.1 for the video-based VR group and 3.4 ±  1.5 for the immersive VR group. Additionally, the response rate for “Remember” was 66.7% for the video-based VR group and 81.7% for the immersive VR group.

**Table 3:** Multiple-choice questions and performance pre- and post-VR operation.

Question	Options (^*^ Correct answer)	Average score and R/K/N of video-based VR group		Average score and R/K/N of immersive VR group	
		Resting phase	Active phase	Resting phase	Active phase
**Q1.** **Select one color for the tourniquet.**	A. Blue B. Green^*^ C. Black D. Transparent	0.2 ± 0.4 Remember: 0 Know: 0 None: 10	0.9 ± 0.3 Remember: 10 Know: 0 None: 0	0.1 ± 0.3 Remember: 0 Know: 0 None: 10	0.8 ± 0.4 Remember: 8 Know: 0 None: 2
**Q2.** **Choose one item used for contamination control.**	A. Protective goggles B. Lead shield C. Disposable gloves^*^ D. Disposable gown	0.8 ± 0.4 Remember: 0 Know: 0 None: 10	1.0 ± 0.0 Remember: 7 Know: 3 None: 0	0.5 ± 0.5 Remember: 0 Know: 0 None: 10	1.0 ± 0.0 Remember: 9 Know: 1 None: 0
**Q3.** **Select the correct action for patient entry and exit.**	A. Independent walking^*^ B. Walking independently with a cane C. Moving using a wheelchair D. Walking with assistance from a caregiver	0.6 ± 0.5 Remember: 0 Know: 0 None: 10	1.0 ± 0.0 Remember: 8 Know: 2 None: 0	0.4 ± 0.5 Remember: 0 Know: 0 None: 10	1.0 ± 0.0 Remember: 9 Know: 0 None: 1
**Q4.** **Identify one action not performed during patient interaction.**	A. Confirming the patient's name B. Checking the patient's condition C. Providing guidance after examination D. Directing the patient to the resting area^*^	0.7 ± 0.5 Remember: 0 Know: 0 None: 10	0.7 ± 0.5 Remember: 4 Know: 3 None: 3	0.3 ± 0.5 Remember: 0 Know: 0 None: 10	0.5 ± 0.5 Remember: 5 Know: 1 None: 4
**Q5.** **Choose one action not performed in RI administration.**	A. Alcohol disinfection B. Puncture at the cubital region C. Connection to an automatic intravenous injector^*^ D. Flushing with normal saline	0.5 ± 0.5 Remember: 0 Know: 0 None: 10	0.9 ± 0.3 Remember: 6 Know: 2 None: 2	0.3 ± 0.5 Remember: 0 Know: 0 None: 10	1.0 ± 0.0 Remember: 10 Know: 0 None: 0
**Q6.** **Select the operation performed on the three-way stopcock.**	A. Air removal in front of the patient B. Labeling of the radiopharmaceutical C. Connection to the infusion line D. Lock operation of a three-way stopcock^*^	0.3 ± 0.5 Remember: 0 Know: 0 None: 10	0.6 ± 0.5 Remember: 5 Know: 2 None: 3	0.1 ± 0.3 Remember: 0 Know: 0 None: 10	0.8 ± 0.4 Remember: 8 Know: 1 None: 1

Six questions were asked, each with four answer options.

* : correct answer. average score, with correct answers of 1 and 0 incorrect answers. Number of responses in each category (remember/know/none).

**Fig 5:**
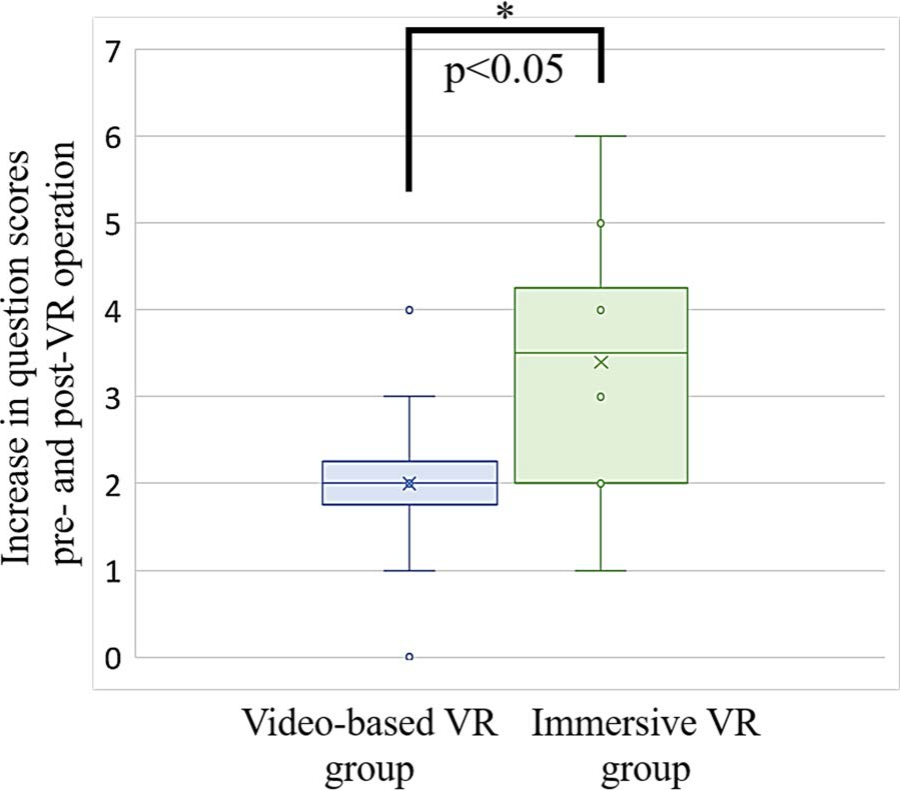
Increase in multiple-choice question scores pre- and post-VR operation.

Fig 5 shows the individual data for the score increase pre- and post-VR operations. Horizontal lines within boxes represent first, median, and third quartiles. Data points exceeding 1.5 times the interquartile range are considered outliers, and the maximum and minimum values are shown. A significant difference in the increase in questionnaire scores between the active and resting phases was found between the video-based and immersive VR groups (p <  0.05).

Box plot showing an increase in the multiple-choice question scores pre- and post-VR. Blue: Video-based VR group; Green: Immersive VR group. Horizontal lines within boxes indicate first, median, and third quartiles. Exclusion of outliers from maximum and minimum values.

## Discussion

### Objective assessment of stress

Biomarkers such as cortisol (a hormone secreted by the adrenal cortex), noradrenaline (a neurotransmitter released from sympathetic nerve terminals), and α-amylase, which is regulated by the sympathetic nervous system, are widely used to assess physiological stress responses [29–30]. Herein, α-amylase was selected as an indicator of sympathetic activity because it is easy to collect from saliva, quickly analyzed, and responds rapidly to stress [31]. An analysis of salivary α-amylase concentration fluctuations pre- and post-VR operations demonstrated a decrease in the video-based VR group (19.2 ±  16.0 KIU/L →  17.4 ±  14.5 KIU/L) and an increasing trend in the immersive VR group (19.9 ±  21.6 KIU/L →  23.0 ±  28.1 KIU/L). These results suggested that immersive VR induces a greater sense of tension. However, these differences were not statistically significant, possibly because of the timing of sample collection. Ideally, saliva should be collected during VR operations; however, owing to technical constraints, saliva was collected within 1 min of the operation. This short interval may have allowed for rapid fluctuations in α-amylase secretion, thereby affecting the results. Additionally, α-amylase secretion varies substantially among individuals, and differences in VR conditions or individual responses may have contributed to the observed variability.

Previous meta-analyses have also indicated that VR exposure does not consistently induce significant changes in α-amylase levels [10]. This underscores the complexity of the relationship between VR exposure and physiological stress responses, suggesting that multiple factors may influence the outcomes. In summary, although α-amylase remains a promising biomarker for the assessment of sympathetic nervous activity, its responses are subject to individual differences and measurement timing. Further research including real-time saliva sampling during VR operations is required to clarify this relationship.

### Subjective mood assessment

VR exposure increases positive emotional states and decreases negative emotional states [32–33]. This hypothesis has been supported by multiple studies highlighting how VR environments can enhance feelings of well-being, happiness, and relaxation while reducing anxiety and stress. Therefore, in this study, we hypothesized that VR exposure influences POMS2 and TDMS-ST responses [34]. However, no significant differences in these measures were observed between the video-based and immersive VR system groups because of two factors. First, we considered the immersion level. Differences in emotional responses are influenced by immersion level [35–36]. However, only a few studies have compared the levels of immersion required for emotional induction. Therefore, it is possible that the differences in immersion between the two VR types were ineffective. Alternatively, if immersive VR was compared to simple two-dimensional video viewing rather than an HMD-based system, the difference in immersion may have been more apparent.

Second, mood scales were evaluated. In this experiment, mood scales were used to observe the degree of tension and concentration exhibited by the participants. These mood scales comprised seven items from the POMS2 and four from the TDMS-ST. However, previous studies have often only focused on evaluating positive and negative emotions. However, even with only two items to evaluate two items, significant individual variation was observed, and previous research reported either no significant or slight differences. Therefore, assessing fluctuations in mood states over short durations by subdividing these scales is challenging. In the future, by focusing solely on positive emotions using tools such as the Positive and Negative Affect Schedule [37–38], we can appropriately evaluate subjective mood scales based on immersion.

In this study, the VR experience lasted 6-10 minutes per participant. While short-term exposure did not cause discomfort, extended use may lead to fatigue or concentration decline. Future research should investigate the impact of prolonged VR use on comfort and task performance. A detailed examination of changes in the ‘Pleasure level’ (comfort) in TDMS-ST pre- and post-VR operation revealed that, although no significant differences were observed, the video-based VR group showed a slight increase (2.00 ±  3.53), whereas the immersive VR group showed a decrease (-1.93 ±  4.11). This suggests a tendency for decreased comfort in immersive VR, indicating that participants may have experienced some level of cognitive or physical load during VR operation. In this study, all participants experienced VR for the first time, and the experimental conditions were standardized across both groups. However, differences in hand size may have influenced the usability of the VR-specific gloves, which could have contributed to the observed discomfort. Although it is difficult to pinpoint the exact cause of the decreased comfort, physical discomfort and cognitive load associated with VR operation may have played a role. To better understand the underlying factors contributing to this decrease in comfort, future studies should incorporate user experience surveys to assess system usability, perceived comfort, and stress responses associated with immersive VR use.

### Objective mood assessment

Reports on the objective evaluation of emotions during VR operations have used metrics such as pulse rate [39], skin conductance and temperature [40], and portable EEG [41]. In this study, we employed a portable EEG to assess tension and focus levels. Giannitrapani et al. investigated the relationship between neural activity and EEG trends during cognitive tasks and reported that β waves (13–30 Hz) are prevalent in states involving thought, focus, problem-solving, logical decision-making, calculations, and mental stress, whereas α waves (8–13 Hz) dominate during relaxed wakefulness and mild focus associated with introspective thought [21]. An increased α/β ratio corresponds to a relaxed and focused state [42], whereas a decreased ratio suggests heightened tension [43].

In this study, the α/β ratio during rest and VR operation (Fig 4) revealed distinct patterns—the video-based VR group experienced a 19% decrease in the ratio, suggesting a state of tension. Conversely, the immersive VR group exhibited a 40% increase in the α/β ratio, indicative of a relaxed and focused mental state. These findings are consistent with previous research showing that a decrease in the α/β ratio is associated with tension, whereas an increase corresponds to a relaxed and concentrated state. Thus, compared with video-based VR systems, immersive VR may provide a more relaxed and focused state during training, promoting the notion that immersive VR enhances the training experience by fostering an optimal mental state, thereby improving focus and learning outcomes. Further research is necessary to confirm these findings and explore the broader impact of VR modalities on the cognitive and emotional states during training.

### Educational effectiveness

As shown in Fig 5, the immersive VR group demonstrated a greater increase in multiple-choice question scores pre- and post-VR operation than the video-based VR group. This finding suggests that immersive VR provides an effective educational experience for training in radiopharmaceutical administration. Additionally, responses to the Remember/Know/None assessment indicated that the immersive VR group had a higher rate of “Remember” responses, implying enhanced memory retention of cognitive functions. These findings align with those of Pande et al., who emphasized the advantages of immersive VR in promoting motivation and enhancing the retention of complex concepts in science learning [44]. The ability of immersive VR to create a deep sense of engagement, particularly with interactive features such as dedicated gloves for finger movements, suggests its potential to improve learning outcomes in medical training. Furthermore, the proposed system allows trainees to progress step-by-step by pressing buttons for each action, which reinforces their memory while making the training more engaging and impactful. Therefore, immersive VR may be a superior platform for effective and memorable training.

**Fig 6:**
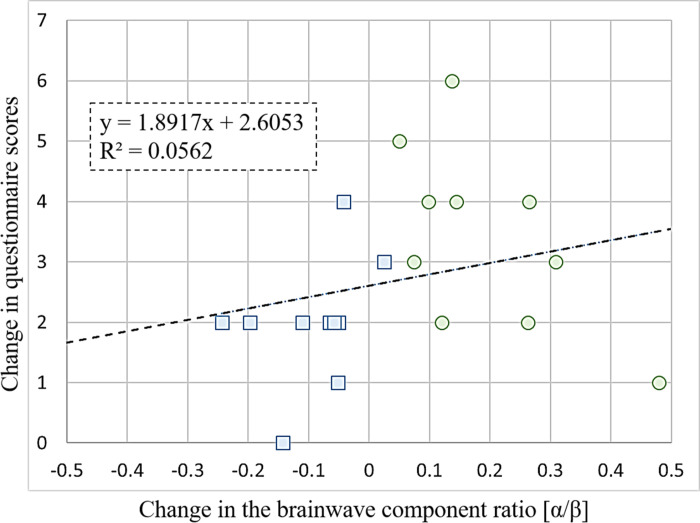
Relationship between the change in α/β ratio and educational effectiveness.

We further investigated the relationship between the α/β ratio and educational effectiveness. In Fig 6, the x-axis represents the change in the α/β ratio between rest and VR operation, while the y-axis shows changes in the number of correct answers on the multiple-choice questions pre- and post-VR operation. The blue and green points represent the video-based and immersive VR groups, respectively, and the dashed line indicates a linear approximation of the two changes using the corresponding equation and correlation coefficient R. The coefficient of determination (R²) was 0.0562, indicating no significant correlation between the α/β ratio and educational effectiveness. However, a negative change in the α/β ratio was associated with a 2.0-point score increase, whereas a positive change was linked to a 3.4-point increase, suggesting that higher α/β values represent a relaxed and focused state, which may enhance learning outcomes. Furthermore, the greater increase in α/β ratio in the immersive VR group suggests that immersive VR may foster better educational effectiveness.

The x-axis shows changes in the brainwave component ratio (α/β), and the y-axis shows the change in questionnaire scores. Blue: Video-based VR group; Green: Immersive VR group. This equation provides a linear approximation of the two changes, where R is the correlation coefficient.

This study evaluated the educational effectiveness of VR training in radiopharmaceutical administration using multiple-choice questionnaires. These results suggest that training in a relaxed and focused state may be effective for improving knowledge retention among students. However, the study did not assess the impact of immersive VR training on the accuracy and safety of medical techniques and procedures. Future research is required to evaluate the educational effects of VR training in improving medical skills and procedural accuracy. In addition, the influence of training in a relaxed and focused state on medical techniques and clinical decision-making under stress in a clinical environment remains unclear. Addressing these issues will help bridge the gap between virtual training and real-world clinical practice, demonstrating the potential value of VR-based training as a tool for the education of diagnostic radiological technologists. Therefore, further studies are needed to explore the connection between a relaxed, focused state and performance in medical training.

### Errors during VR operation

In this study, we implemented a medical support system to mitigate VR sickness and nausea. However, none of the participants reported any symptoms or requested to discontinue the experiment. Furthermore, technical issues such as interference from the wired connection of the head-mounted display or missing EEG data were addressed by excluding affected samples (Fig 2). Therefore, all the collected data were obtained under consistent conditions.

There were no significant deviations in the VR operation from the experimental procedures. Because the system was event-driven, it did not automatically transition to the next action, allowing the participants to follow the correct procedure step-by-step by reading the instructions on the button and performing the corresponding action. The vibration feedback feature of the VR gloves was set to respond only when the participants correctly grasped the objects. In addition, providing feedback via vibrations when incorrect techniques or procedures are performed can help reinforce the learning of correct actions. The incorporation of error detection functions, such as those used in airborne tactile technology, has been shown to be useful in grasping radiation distribution in medical contexts, as reported in previous studies [45]. This error detection function may improve the safety and educational effectiveness of training in the handling and administration of radiopharmaceuticals.

### Limitations

This study has several limitations. A portable EEG device was used to reduce interference from the HMD. However, because data were extracted only from the p1, Fpz, and Fp2 sites in the international 10-20 system, using a medical-grade EEG device would have provided more accurate data. A comparison of EEG data from simple and medical-grade devices during VR operation is planned for future research.

Another limitation concerns the VR systems. The prototype VR system developed for training radiological technologists in radiopharmaceutical administration does not fully replicate sensations, such as needle insertion or extension tube flexibility. Additionally, although dedicated gloves are used to project hand movements onto the VR space, differences in hand size may affect movement sensations. Future developments should integrate augmented reality with virtual environments for better comparison with real-world medical training. Nevertheless, the immersive VR system demonstrated educational effectiveness, indicating its potential value for student training.

This study primarily targeted university students around the age of 20 years, specifically first- and second-year students enrolled in a radiological technologist training program before they underwent clinical practice. Because of its focus on this specific group, the study did not include participants who were experienced technologists with varying years of professional experience. Consequently, the effects of VR training across different levels of experience have not been directly compared. Moreover, there were no reports in our study evaluating the effectiveness of VR training based on proficiency levels. Given that experience level may influence how technologists respond to training, future research will aim to address this gap by considering differences in years of professional experience. By including a broader range of participants with various levels of expertise, future studies should conduct comparative analyses to assess how VR training systems may affect both novice and experienced technologists.

Another limitation of this study is the lack of clarity regarding the stress factors experienced by participants. The study design incorporated various factors such as the stress caused by pure VR operation, wearing the hardware, time pressure, task complexity, and adaptation to the VR environment. All of these factors were measured collectively. Future research should isolate these factors individually to specifically evaluate the stress caused by VR operations alone. Additionally, although no significant differences in salivary amylase levels were observed pre- and post-VR operation in this study, it remains unclear whether this result indicates the absence of physiological stress, as indicated by salivary amylase, or whether the timing of the measurement points influenced this result. Nida Ali et al. suggest that salivary alpha-amylase is a useful tool in behavioral medicine and can be an effective biomarker for evaluating psychological stress, particularly in non-invasive settings [46]. However, individual differences in salivary alpha-amylase levels can be substantial, which highlights the importance of ensuring an adequate sample size. Furthermore, the timing of measurements is critical, and standardization is necessary to improve reliability. Combining salivary alpha-amylase measurements with other biomarkers, such as heart rate or cortisol, may enhance the accuracy of stress assessments [46, 47]. It is also possible that the immediate responsiveness of amylase and the timing of the measurements did not align with the stress response induced by VR training [31]. Further research is required to address these issues and clarify the factors that contribute to the physiological stress response.

In addition, although this study demonstrated the educational effectiveness of immersive VR compared to video-based VR, further investigation is required to evaluate its contribution to the acquisition of actual medical skills. The current study did not compare VR training with hands-on practice using real equipment, making it unclear whether VR serves as a complementary tool or a viable alternative to traditional training methods. Recent research has increasingly focused on the comparison between VR-based training and traditional hands-on methods. In the field of laparoscopic surgery training, studies have demonstrated that VR-based training significantly improves operating room performance compared to conventional methods, leading to fewer errors and reduced procedure time [48]. A scoping review on the applications of virtual reality in healthcare training suggests that VR can enhance learning outcomes, yet emphasizes the need for direct comparisons with hands-on practice to establish its role in medical education [49]. While studies in dental education have demonstrated the educational benefits of VR, systematic reviews indicate that further research is needed to fully understand its effectiveness relative to conventional training [50]. On the other hand, in the field of radiation sciences, such comparative studies remain limited. Given the unique nature of radiation-related procedures, assessing the applicability of VR-based training in this domain is crucial. To address this limitation, our next research phase will involve evaluating medical skill acquisition in both real and virtual environments. If VR-based training proves to be as effective as real-world practice, it could serve as a valuable training tool for students, offering repeated training opportunities without the risk of radiation exposure or puncture injury. Future studies should compare VR training with real-world hands-on practice to determine whether VR can replace or effectively supplement conventional training methods.

## Conclusion

Two VR systems, video-based and immersive, were designed for training in radiopharmaceutical administration, a task expanded to include radiological technologists owing to task shifting. Subjective mood assessments using POMS2 and TDMS-ST revealed no significant differences between the two systems. Although salivary amylase levels showed no significant differences, immersive VR tended to induce a slightly higher tension. However, the EEG analysis revealed that immersive VR induced a more relaxed and focused state and demonstrated better educational outcomes than video-based VR. Thus, immersive VR enhanced the effectiveness of student training in radiopharmaceutical administration. Future studies should incorporate medical skill assessments to evaluate the proficiency of this technique.

## Supporting information

S1 Table Subject demographics.
(DOCX)

S2 Table Salivary amylase levels pre- and post-VR operation.
(DOCX)

S3 Table POMS2 score pre- and post-VR operation.
(DOCX)

S4 Table TDMS-ST score pre- and post-VR operation.
(DOCX)

S5 Table α/β ratio pre- and post-VR operation.
(DOCX)

S6 Table Multiple-choice questions and performance pre- and post-VR operation.
(DOCX)

## References

[1] Ministry of Health, Labour and Welfare. Revision of the medical care act and other related laws to promote the establishment of a system for efficiently providing high-quality and appropriate medical care. [Cited 2024 Nov 18]. Available from: https://www.mhlw.go.jp/stf/seisakunitsuite/bunya/kenkou_iryou/iryou/ishi-hatarakikata_34355.html

[2] Harris S, Crowley JR, Warden N. Radiopharmaceutical administration practices-Are they best practice?. Front Nucl Med. 2023;3:1244660. doi: 10.3389/fnume.2023.1244660 39355051 PMC11440992

[3] Guo Y, Mao L, Zhang G, Chen Z, Pei X, Xu XG. Conceptual design and preliminary results of a vr-based radiation safety training system for interventional radiologists. Radiat Prot Dosimetry. 2020;190(1):58–65. doi: 10.1093/rpd/ncaa082 32501514

[4] Nishi K, Fujibuchi T, Yoshinaga T. Development and evaluation of the effectiveness of educational material for radiological protection that uses augmented reality and virtual reality to visualise the behaviour of scattered radiation. J Radiol Prot. 2022;42(1):10.1088/1361-6498/ac3e0a. doi: 10.1088/1361-6498/ac3e0a 34844224

[5] Rainford L, Tcacenco A, Potocnik J, Brophy C, Lunney A, Kearney D, et al. Student perceptions of the use of three-dimensional (3-D) virtual reality (VR) simulation in the delivery of radiation protection training for radiography and medical students. Radiography (Lond). 2023;29(4):777–85. doi: 10.1016/j.radi.2023.05.009 37244141

[6] Leek A, Keren N, Lawson A, Webster A. Using Non-immersive VR Simulations in Conjunction with Priming to Enhance Conceptualizing Radiation and Risk. Interserv Ind Train Simul Educ Conf. 2023;1(23174):1–11. 38094077 PMC10716861

[7] Khamruang S, Marshall S, Sirieak N, Karnkorn P, Keawtong V, Hayeeabdunromae A, et al. Nuclear medicine radiological hot laboratory simulation: A mixed-method intervention study on immersive virtual reality for sustainable education. Applied Sciences. 2024;14(12):5041.

[8] Meese MM, O’Hagan EC, Chang TP. Healthcare provider stress and virtual reality simulation: a scoping review. Simul Healthc. 2021;16(4):268–74. doi: 10.1097/SIH.0000000000000484 32890319

[9] Maruyama Y, Kawano A, Okamoto S, Ando T, Ishitobi Y, Tanaka Y, et al. Differences in salivary alpha-amylase and cortisol responsiveness following exposure to electrical stimulation versus the Trier Social Stress Tests. PLoS One. 2012;7(7):e39375. doi: 10.1371/journal.pone.0039375 22859941 PMC3408464

[10] Dammen L van, Finseth TT, McCurdy BH, Barnett NP, Conrady RA, Leach AG, et al. Evoking stress reactivity in virtual reality: A systematic review and meta-analysis. Neurosci Biobehav Rev. 2022;138:104709. doi: 10.1016/j.neubiorev.2022.104709 35644278

[11] Abu Hasan R, Sulaiman S, Ashykin NN, Abdullah MN, Hafeez Y, Ali SSA. Workplace mental state monitoring during VR-based training for offshore environment. Sensors (Basel). 2021;21(14):4885. doi: 10.3390/s21144885 34300624 PMC8309835

[12] Jo S-H, Park J-S, Yeon P-S. The effect of forest video using virtual reality on the stress reduction of university students focused on C university in Korea. Int J Environ Res Public Health. 2021;18(23):12805. doi: 10.3390/ijerph182312805 34886531 PMC8657194

[13] Shacham S. A shortened version of the profile of mood states. J Pers Assess. 1983;47(3):305–6. doi: 10.1207/s15327752jpa4703_14 6886962

[14] Kuboki T, Nomura S, Wada M, Akabayashi A, Nagataki M, Suematsu H, et al. Multidimensional assessment of mental state in occupational health care--combined application of three questionnaires: Tokyo University Egogram (TEG), Time Structuring Scale (TSS), and Profile of Mood States (POMS). Environ Res. 1993;61(2):285–98. doi: 10.1006/enrs.1993.1073 8495670

[15] Rossi V, Pourtois G. Transient state-dependent fluctuations in anxiety measured using STAI, POMS, PANAS or VAS: a comparative review. Anxiety Stress Coping. 2012;25(6):603–45. doi: 10.1080/10615806.2011.582948 21827372

[16] KONUMA H, HIROSE H, YOKOYAMA K. Relationship of the Japanese Translation of the Profile of Mood States Second Edition (POMS 2&reg;) to the First Edition (POMS&reg;). Juntendo Medical Journal. 2015;61(5):517–9. doi: 10.14789/jmj.61.517

[17] Matsui Y, Ueda T, Koizumi Y, Kato C, Suzuki Y. Crossover trial of the effects of a far-infrared heater that heats the feet with ceramic balls on autonomic nervous activity and mood states. Sci Prog. 2023;106(1):368504231158452. doi: 10.1177/00368504231158452 36862583 PMC10450299

[18] Sakairi Y, Nakatsuka K, Shimizu T. Development of the two-dimensional mood scale for self-monitoring and self-regulation of momentary mood states. Jpn Psychol Res. 2013;55:338–49.

[19] Tulving E. Memory and consciousness. Canadian Psychologist. 1985;26(1):1–12.

[20] Rajaram S. Remembering and knowing: two means of access to the personal past. Mem Cognit. 1993;21(1):89–102. doi: 10.3758/bf03211168 8433652

[21] Giannitrapani D, Murri L. The EEG of Mental Activities. Karger. 1988;149–52.

[22] Wen T, Aris S. Electroencephalogram (EEG) stress analysis on alpha/beta ratio and theta/beta ratio. Indones J Electr Eng Comput Sci. 2020;17(1):175–82.

[23] You SD. Classification of Relaxation and Concentration Mental States with EEG. Information. 2021;12(5):187. doi: 10.3390/info12050187

[24] Likert R. A technique for the measurement of attitudes. Archives of Psychology. 1932;140(1):1–55.

[25] Schulz JB, Dubrowski P, Blomain E, Million L, Qian Y, Marquez C, et al. An Affordable Platform for Virtual Reality-Based Patient Education in Radiation Therapy. Pract Radiat Oncol. 2023;13(6):e475–83. doi: 10.1016/j.prro.2023.06.008 37482182

[26] Samadbeik M, Yaaghobi D, Bastani P, Abhari S, Rezaee R, Garavand A. The applications of virtual reality technology in medical groups teaching. J Adv Med Educ Prof. 2018;6(3):123–9. 30013996 PMC6039818

[27] Hussain Z, Ng DM, Alnafisee N, Sheikh Z, Ng N, Khan A, et al. Effectiveness of virtual and augmented reality for improving knowledge and skills in medical students: protocol for a systematic review. BMJ Open. 2021;11(8):e047004. doi: 10.1136/bmjopen-2020-047004 34400451 PMC8370502

[28] Steen CW, Söderström K, Stensrud B, Nylund IB, Siqveland J. The effectiveness of virtual reality training on knowledge, skills and attitudes of health care professionals and students in assessing and treating mental health disorders: a systematic review. BMC Med Educ. 2024;24(1):480. doi: 10.1186/s12909-024-05423-0 38693509 PMC11064237

[29] Speirs RL, Herring J, Cooper WD, Hardy CC, Hind CR. The influence of sympathetic activity and isoprenaline on the secretion of amylase from the human parotid gland. Arch Oral Biol. 1974;19(9):747–52. doi: 10.1016/0003-9969(74)90161-7 4533726

[30] Yamaguchi M, Kanemori T, Kanemaru M, Takai N, Mizuno Y, Yoshida H. Performance evaluation of salivary amylase activity monitor. Biosens Bioelectron. 2004;20(3):491–7. doi: 10.1016/j.bios.2004.02.012 15494230

[31] Takai N, Yamaguchi M, Aragaki T, Eto K, Uchihashi K, Nishikawa Y. Effect of psychological stress on the salivary cortisol and amylase levels in healthy young adults. Arch Oral Biol. 2004;49(12):963–8. doi: 10.1016/j.archoralbio.2004.06.007 15485637

[32] Pavic K, Vergilino-Perez D, Gricourt T, Chaby L. Because I’m happy—an overview on fostering positive emotions through virtual reality. Frontiers in Virtual Reality. 2022;3 788820. doi: 10.3389/frvir.2022.788820

[33] Ślósarz L, Jurczyk-Romanowska E, Rosińczuk J, Kazimierska-Zając M. Virtual reality as a teaching resource which reinforces emotions in the teaching process. Sage Open. 2022;12(3):. doi: 10.1177/21582440221118083

[34] Ochi G, Kuwamizu R, Fujimoto T, Ikarashi K, Yamashiro K, Sato D. The effects of acute virtual reality exergaming on mood and executive function: exploratory crossover trial. JMIR Serious Games. 2022;10(3):e38200. doi: 10.2196/38200 36169992 PMC9557761

[35] Visch VT, Tan ES, Molenaar D. The emotional and cognitive effect of immersion in film viewing. Cogn Emot. 2010;24:1439–45.

[36] Diemer J, Alpers GW, Peperkorn HM, Shiban Y, Mühlberger A. The impact of perception and presence on emotional reactions: a review of research in virtual reality. Front Psychol. 2015;6:26. doi: 10.3389/fpsyg.2015.00026 25688218 PMC4311610

[37] Zulkarnain AHB, Cao X, Kókai Z, Gere A. Self-Assessed Experience of Emotional Involvement in Sensory Analysis Performed in Virtual Reality. Foods. 2024;13(3):375. doi: 10.3390/foods13030375 38338511 PMC10855596

[38] Watson D, Clark LA, Tellegen A. Development and validation of brief measures of positive and negative affect: the PANAS scales. J Pers Soc Psychol. 1988;54(6):1063–70. doi: 10.1037//0022-3514.54.6.1063 3397865

[39] Oliveira T, Noriega P, Carvalhais J, Rebelo F, Lameira V. How deep is a virtual reality experience? Virtual environments, emotions and physiological measures. In: Rebelo F, Soares MM, editors. Advances in ergonomics in design. Advances in intelligent systems and computing. Cham: Springer International Publishing; 2020 p. 462–71.

[40] Quazi M, Mukhopadhyay S, Suryadevara N, Huang Y. Towards the smart sensors based human emotion recognition. 2012 IEEE International Instrumentation and Measurement Technology Conference Proceedings. 2012; p. 2365–70.

[41] Suhaimi N, Teo J, Mountstephens J. Empirical analysis of intra vs. inter-subject variability in VR EEG-based emotion modelling. J Eng Appl Sci. 2018;13(8):2137–44.

[42] Kim S, Park H, Choo S. Effects of changes to architectural elements on human relaxation-arousal responses: based on VR and EEG. Int J Environ Res Public Health. 2021;18(8):4305. doi: 10.3390/ijerph18084305 33921601 PMC8074029

[43] Griffiths BJ, Mayhew SD, Mullinger KJ, Jorge J, Charest I, Wimber M, et al. Alpha/beta power decreases track the fidelity of stimulus-specific information. Elife. 2019;8 e49562. doi: 10.7554/eLife.49562 31782730 PMC6904219

[44] Pande P, Thit A, Sørensen A, Mojsoska B, Moeller M, Jepsen P. Long-term effectiveness of immersive VR simulations in undergraduate science learning: lessons from a media-comparison study. Research in Learning Technology. 2021;29:2482.

[45] Fujise D, Koba Y, Hasegawa S, Negishi T. Development and Evaluation of Educational Systems Using Airborne Tactile Technology for Medical Radiation Protection. Sensors and Materials. 2025;37(1):63. doi: 10.18494/sam5418

[46] Ali N, Nater UM. Salivary Alpha-Amylase as a Biomarker of Stress in Behavioral Medicine. Int J Behav Med. 2020;27(3):337–42. doi: 10.1007/s12529-019-09843-x 31900867 PMC7250801

[47] Nater UM, La Marca R, Florin L, Moses A, Langhans W, Koller MM, et al. Stress-induced changes in human salivary alpha-amylase activity -- associations with adrenergic activity. Psychoneuroendocrinology. 2006;31(1):49–58. doi: 10.1016/j.psyneuen.2005.05.010 16002223

[48] Seymour NE, Gallagher AG, Roman SA, O’Brien MK, Bansal VK, Andersen DK, et al. Virtual reality training improves operating room performance: results of a randomized, double-blinded study. Ann Surg. 2002;236(4):458–63; discussion 463-4. doi: 10.1097/00000658-200210000-00008 12368674 PMC1422600

[49] Philip G, Savundranayagam MY. Applications of virtual reality and its effectiveness in healthcare training: a scoping review. Health Technol. 2024. doi: 10.1007/s12553-024-00936-6

[50] Koolivand H, Shooreshi MM, Safari-Faramani R, Borji M, Mansoory MS, Moradpoor H, et al. Comparison of the effectiveness of virtual reality-based education and conventional teaching methods in dental education: a systematic review. BMC Med Educ. 2024;24(1):8. doi: 10.1186/s12909-023-04954-2 38172742 PMC10765860

