# Supplementary material for: Virtual reality-based training for radiopharmaceutical administration: development and educational effectiveness
Source: PLoS One. 2025 Mar 31;20(3):e0321101. doi: 10.1371/journal.pone.0321101 (PMC11957288; doi:10.1371/journal.pone.0321101)
Supplement: S1 Table — (DOCX) [file pone.0321101.s001.docx]

**Supplementary information**

**S1 Table1.** **Subject demographics**

| Video-based VR group | | | Immersive VR group | | |
| --- | --- | --- | --- | --- | --- |
| Subject No. | Age | Sex | Subject No. | Age | Sex |
| V01 | 21 | Male | I01 | 21 | Male |
| V02 | 21 | Male | I02 | 20 | Female |
| V03 | 21 | Male | I03 | 21 | Female |
| V04 | 21 | Female | I04 | 20 | Male |
| V05 | 18 | Female | I05 | 22 | Male |
| V06 | 20 | Female | I06 | 20 | Female |
| V07 | 20 | Male | I07 | 19 | Male |
| V08 | 21 | Male | I08 | 21 | Male |
| V09 | 19 | Male | I09 | 20 | Male |
| V10 | 20 | Male | I10 | 19 | Female |
| V11 | 20 | Male | I11 | 19 | Female |
| V12 | 21 | Male | I12 | 19 | Female |
| V13 | 20 | Female | I13 | 19 | Female |
| V14 | 21 | Male | I14 | 19 | Female |
|  |  |  | I15 | 20 | Male |
