# Supplementary material for: Virtual reality-based training for radiopharmaceutical administration: development and educational effectiveness
Source: PLoS One. 2025 Mar 31;20(3):e0321101. doi: 10.1371/journal.pone.0321101 (PMC11957288; doi:10.1371/journal.pone.0321101)
Supplement: S2 Table — (DOCX) [file pone.0321101.s002.docx]

**Supplementary information**

**S2 Table1. Salivary Amylase Levels pre- and post-VR operation**

| Video-based VR group | | | Immersive VR group | | |
| --- | --- | --- | --- | --- | --- |
| Subject No. | Salivary Amylase Levels [KIU/L] | | Subject No. | Salivary Amylase Levels [KIU/L] | |
|  | Pre-VR | Post-VR |  | Pre-VR | Post-VR |
| V01 | 40 | 16 | I01 | 36 | 58 |
| V02 | 53 | 11 | I02 | 31 | 9 |
| V03 | 7 | 29 | I03 | 4 | 4 |
| V04 | 45 | 33 | I04 | 4 | 4 |
| V05 | 3 | 6 | I05 | 36 | 38 |
| V06 | 8 | 4 | I06 | 20 | 4 |
| V07 | 14 | 14 | I07 | 85 | 104 |
| V08 | 23 | 5 | I08 | 12 | 18 |
| V09 | 6 | 3 | I09 | 7 | 5 |
| V10 | 17 | 5 | I10 | 4 | 4 |
| V11 | 16 | 10 | I11 | 4 | 18 |
| V12 | 3 | 45 | I12 | 7 | 4 |
| V13 | 21 | 43 | I13 | 4 | 4 |
| V14 | 13 | 20 | I14 | 21 | 35 |
|  |  |  | I15 | 23 | 36 |
