# Supplementary material for: Virtual reality-based training for radiopharmaceutical administration: development and educational effectiveness
Source: PLoS One. 2025 Mar 31;20(3):e0321101. doi: 10.1371/journal.pone.0321101 (PMC11957288; doi:10.1371/journal.pone.0321101)
Supplement: S3 Table — (DOCX) [file pone.0321101.s003.docx]

**Supplementary information**

**S3 Table.** **POMS2 score pre- and post-VR operation**

| Video-based VR group | | | | | | | | | | | | | | | | |
| --- | --- | --- | --- | --- | --- | --- | --- | --- | --- | --- | --- | --- | --- | --- | --- | --- |
| Subject  No | Pre-VR | | | | | | | | Post-VR | | | | | | | |
|  | AH | CB | DD | FI | TA | VA | F | TMD | AH | CB | DD | FI | TA | VA | F | TMD |
| V01 | 0 | 0 | 0 | 0 | 0 | 0 | 0 | 0 | 0 | 1 | 0 | 4 | 1 | 0 | 3 | 6 |
| V02 | 8 | 10 | 12 | 6 | 10 | 2 | 7 | 44 | 3 | 7 | 3 | 5 | 11 | 8 | 10 | 21 |
| V03 | 0 | 0 | 4 | 0 | 4 | 16 | 15 | -8 | 0 | 0 | 0 | 0 | 1 | 20 | 20 | -19 |
| V04 | 2 | 3 | 3 | 7 | 3 | 7 | 11 | 11 | 0 | 4 | 2 | 5 | 3 | 6 | 9 | 8 |
| V05 | 8 | 20 | 13 | 17 | 12 | 16 | 14 | 54 | 6 | 17 | 8 | 19 | 14 | 12 | 18 | 52 |
| V06 | 5 | 16 | 14 | 14 | 15 | 3 | 13 | 61 | 5 | 13 | 18 | 11 | 16 | 5 | 13 | 58 |
| V07 | 2 | 11 | 7 | 13 | 7 | 11 | 14 | 29 | 0 | 6 | 2 | 6 | 4 | 11 | 17 | 7 |
| V08 | 3 | 7 | 6 | 4 | 9 | 10 | 13 | 19 | 1 | 7 | 5 | 4 | 6 | 8 | 12 | 15 |
| V09 | 0 | 3 | 7 | 6 | 9 | 11 | 16 | 14 | 0 | 2 | 0 | 2 | 3 | 11 | 16 | -4 |
| V10 | 6 | 18 | 14 | 15 | 14 | 2 | 17 | 65 | 2 | 10 | 3 | 11 | 11 | 10 | 16 | 27 |
| V11 | 0 | 0 | 0 | 3 | 4 | 5 | 10 | 2 | 0 | 0 | 0 | 4 | 3 | 5 | 14 | 2 |
| V12 | 0 | 5 | 2 | 8 | 5 | 8 | 9 | 12 | 8 | 5 | 7 | 14 | 9 | 6 | 8 | 37 |
| V13 | 2 | 1 | 6 | 0 | 3 | 2 | 6 | 10 | 1 | 0 | 1 | 0 | 0 | 0 | 4 | 2 |
| V14 | 2 | 10 | 8 | 4 | 12 | 12 | 13 | 24 | 0 | 9 | 1 | 4 | 10 | 10 | 11 | 14 |
| Immersive VR group | | | | | | | | | | | | | | | | |
| Subject  No | Pre-VR | | | | | | | | Post-VR | | | | | | | |
|  | AH | CB | DD | FI | TA | VA | F | TMD | AH | CB | DD | FI | TA | VA | F | TMD |
| I01 | 0 | 1 | 0 | 0 | 2 | 13 | 9 | -10 | 0 | 3 | 0 | 4 | 5 | 5 | 7 | 7 |
| I02 | 0 | 1 | 0 | 0 | 8 | 4 | 11 | 5 | 0 | 1 | 0 | 2 | 0 | 6 | 9 | -3 |
| I03 | 6 | 10 | 7 | 10 | 11 | 11 | 13 | 33 | 9 | 10 | 9 | 8 | 11 | 8 | 12 | 39 |
| I04 | 4 | 7 | 5 | 9 | 5 | 11 | 12 | 19 | 6 | 7 | 5 | 10 | 7 | 10 | 11 | 25 |
| I05 | 5 | 5 | 3 | 3 | 9 | 5 | 9 | 20 | 1 | 8 | 7 | 5 | 5 | 10 | 10 | 16 |
| I06 | 2 | 2 | 1 | 1 | 2 | 8 | 13 | 0 | 0 | 0 | 0 | 0 | 0 | 9 | 12 | -9 |
| I07 | 0 | 0 | 0 | 0 | 2 | 2 | 8 | 0 | 0 | 0 | 0 | 0 | 0 | 1 | 4 | -1 |
| I08 | 0 | 2 | 4 | 2 | 1 | 12 | 13 | -3 | 0 | 5 | 3 | 2 | 2 | 10 | 14 | 2 |
| I09 | 0 | 1 | 0 | 7 | 2 | 10 | 13 | 0 | 0 | 0 | 0 | 4 | 0 | 10 | 13 | -6 |
| I10 | 0 | 1 | 0 | 3 | 3 | 10 | 13 | -3 | 0 | 0 | 0 | 1 | 0 | 6 | 13 | -5 |
| I11 | 6 | 8 | 9 | 8 | 7 | 9 | 9 | 29 | 5 | 5 | 7 | 6 | 5 | 5 | 10 | 23 |
| I12 | 0 | 4 | 0 | 5 | 6 | 9 | 11 | 6 | 0 | 1 | 0 | 1 | 2 | 9 | 9 | -5 |
| I13 | 3 | 3 | 1 | 3 | 8 | 11 | 16 | 7 | 0 | 0 | 0 | 1 | 3 | 12 | 14 | -8 |
| I14 | 0 |  | 0 | 0 | 0 | 4 | 4 | -3 | 0 | 0 | 0 | 0 | 1 | 3 | 3 | -2 |
| I15 | 0 | 0 | 0 | 0 | 0 | 19 | 15 | -19 | 0 | 3 | 0 | 1 | 4 | 16 | 18 | -8 |

POMS2: profile of mood states in the second edition. Mood scale items: anger, hostility (AH); confusion, bewilderment (CB); depression, dejection (DD); fatigue, inertia (FI); tension, anxiety (TA); vigorous, activity (VA); and friendliness (F), total mood disturbance (TMD).
