# Supplementary material for: Virtual reality-based training for radiopharmaceutical administration: development and educational effectiveness
Source: PLoS One. 2025 Mar 31;20(3):e0321101. doi: 10.1371/journal.pone.0321101 (PMC11957288; doi:10.1371/journal.pone.0321101)
Supplement: S4 Table — (DOCX) [file pone.0321101.s004.docx]

**Supplementary information**

**S4 Table.** **TDMS-ST score pre- and post-VR operation**

| Video-based VR group | | | | | | | | | | | | | | | |
| --- | --- | --- | --- | --- | --- | --- | --- | --- | --- | --- | --- | --- | --- | --- | --- |
| Subject  No | Pre-VR | | | | | | | Post-VR | | | | | | | |
|  | V | S | | P | | A | | V | | S | | P | | A | |
| V01 | 1 | 6 | | 7 | | -5 | | 0 | | 4 | | 4 | | -4 | |
| V02 | -2 | 1 | | -1 | | -3 | | 4 | | 3 | | 7 | | 1 | |
| V03 | 10 | 10 | | 20 | | 0 | | 10 | | 10 | | 20 | | 0 | |
| V04 | 0 | 5 | | 5 | | -5 | | 2 | | 4 | | 6 | | -2 | |
| V05 | -1 | 10 | | 9 | | -11 | | 2 | | 10 | | 12 | | -8 | |
| V06 | -3 | 6 | | 3 | | -9 | | -2 | | 7 | | 5 | | -9 | |
| V07 | -4 | 10 | | 6 | | -14 | | 4 | | 7 | | 11 | | -3 | |
| V08 | 2 | 7 | | 9 | | -5 | | 3 | | 7 | | 10 | | -4 | |
| V09 | 5 | 4 | | 9 | | 1 | | 3 | | 6 | | 9 | | -3 | |
| V10 | -8 | 7 | | -1 | | -15 | | -1 | | 10 | | 9 | | -11 | |
| V11 | 2 | 3 | | 5 | | -1 | | 1 | | 3 | | 4 | | -2 | |
| V12 | -1 | 5 | | 4 | | -6 | | 0 | | 4 | | 4 | | -4 | |
| V13 | 0 | 2 | | 2 | | -2 | | 0 | | 2 | | 2 | | -2 | |
| V14 | 3 | 4 | | 7 | | -1 | | 5 | | 4 | | 9 | | 1 | |
| Immersive VR group | | | | | | | | | | | | | | | |
| Subject  No | Pre-VR | | | | | | | | Post-VR | | | | | | |
|  | V | | S | | P | | A | | V | | S | | P | | A |
| I01 | 3 | | 2 | | 5 | | 1 | | 1 | | 1 | | 2 | | 0 |
| I02 | 3 | | 3 | | 6 | | 0 | | 2 | | 2 | | 4 | | 0 |
| I03 | -2 | | 4 | | 2 | | -6 | | -1 | | 4 | | 3 | | -5 |
| I04 | -1 | | 7 | | 6 | | -8 | | 1 | | 6 | | 7 | | -5 |
| I05 | 1 | | 4 | | 5 | | -3 | | 3 | | 2 | | 5 | | 1 |
| I06 | 6 | | 9 | | 15 | | -3 | | 7 | | 10 | | 17 | | -3 |
| I07 | 3 | | 8 | | 11 | | -5 | | 1 | | 3 | | 4 | | -2 |
| I08 | 4 | | 7 | | 11 | | -3 | | 4 | | 1 | | 5 | | 3 |
| I09 | -5 | | 6 | | 1 | | -11 | | -4 | | 6 | | 2 | | -10 |
| I10 | 2 | | 6 | | 8 | | -4 | | 2 | | 5 | | 7 | | -3 |
| I11 | 0 | | 8 | | 8 | | -8 | | 0 | | 9 | | 9 | | -9 |
| I12 | 4 | | 5 | | 9 | | -1 | | 3 | | 4 | | 7 | | -1 |
| I13 | 4 | | 7 | | 11 | | -3 | | 6 | | 7 | | 13 | | -1 |
| I14 | 4 | | 10 | | 14 | | -6 | | 1 | | 10 | | 11 | | -9 |
| I15 | 10 | | 8 | | 18 | | 2 | | 4 | | 1 | | 5 | | 3 |

TDMS-ST: two-dimensional mood scale-short term. Mood scale items: vitality (V), stability (S), pleasure (P), and arousal (A)
