# Supplementary material for: Virtual reality-based training for radiopharmaceutical administration: development and educational effectiveness
Source: PLoS One. 2025 Mar 31;20(3):e0321101. doi: 10.1371/journal.pone.0321101 (PMC11957288; doi:10.1371/journal.pone.0321101)
Supplement: S5 Table — (DOCX) [file pone.0321101.s005.docx]

**Supplementary information**

**S5 Table.** **α/β ratio pre- and post-VR operation**

| Video-based VR group | | | Immersive VR group | | |
| --- | --- | --- | --- | --- | --- |
| Subject No. | α/β ratio | | Subject No. | α/β ratio | |
|  | Pre-VR | Post-VR |  | Pre-VR | Post-VR |
| V01 | 0.419 | 0.387 | I01 | 0.513 | 0.436 |
| V02 | 0.353 | 0.285 | I02 | 0.483 | 0.507 |
| V03 | 0.265 | 0.267 | I03 | 0.221 | 0.293 |
| V04 | 0.337 | 0.243 | I04 | 0.222 | 0.648 |
| V05 | 0.257 | 0.282 | I05 | 0.623 | 0.740 |
| V06 | 0.538 | 0.487 | I06 | 0.518 | 0.781 |
| V07 | 0.401 | 0.335 | I07 | 0.392 | 0.466 |
| V08 | 0.339 | 0.288 | I08 | 0.445 | 0.753 |
| V09 | 0.633 | 0.490 | I09 | 0.279 | 0.399 |
| V10 | 0.492 | 0.381 | I10 | 0.435 | 0.579 |
| V11 | 0.282 | 0.240 | I11 | 0.240 | 0.719 |
| V12 | 0.714 | 0.517 | I12 | 0.568 | 0.617 |
| V13 | 0.764 | 0.520 | I13 | 0.314 | 0.579 |
| V14 | 0.287 | 0.229 | I14 | 0.381 | 0.518 |
|  |  |  | I15 | 0.644 | 0.742 |
