# Supplementary material for: Virtual reality-based training for radiopharmaceutical administration: development and educational effectiveness
Source: PLoS One. 2025 Mar 31;20(3):e0321101. doi: 10.1371/journal.pone.0321101 (PMC11957288; doi:10.1371/journal.pone.0321101)
Supplement: S6 Table — (DOCX) [file pone.0321101.s006.docx]

**Supplementary information**

**S6 Table.** **Multiple-choice questions and performance pre- and post-VR operation**

| Video-based VR group | | | | | | | | | | | | | | | | | | | | | | | | |
| --- | --- | --- | --- | --- | --- | --- | --- | --- | --- | --- | --- | --- | --- | --- | --- | --- | --- | --- | --- | --- | --- | --- | --- | --- |
| Subject  No | Pre-VR (multiple-choice questions) | | | | | | Pre-VR (remember[R] / know [K] / none [N]) | | | | | | Post-VR (multiple-choice questions) | | | | | | Post-VR (remember[R] / know [K] / none [N]) | | | | | |
|  | Q1 | Q2 | Q3 | Q4 | Q5 | Q6 | RKN1 | RKN2 | RKN3 | RKN4 | RKN5 | RKN6 | Q1 | Q2 | Q3 | Q4 | Q5 | Q6 | RKN1 | RKN2 | RKN3 | RKN4 | RKN5 | RKN6 |
| V05 | 0 | 0 | 0 | 1 | 0 | 0 | N | N | N | N | N | N | 1 | 1 | 1 | 0 | 1 | 0 | R | R | R | R | R | R |
| V06 | 0 | 1 | 1 | 1 | 1 | 0 | N | N | N | N | N | N | 1 | 1 | 1 | 1 | 1 | 1 | R | R | R | K | R | K |
| V07 | 1 | 0 | 1 | 1 | 1 | 0 | N | N | N | N | N | N | 1 | 1 | 1 | 1 | 1 | 1 | R | R | K | K | K | R |
| V08 | 0 | 1 | 1 | 1 | 1 | 1 | N | N | N | N | N | N | 1 | 1 | 1 | 1 | 1 | 1 | R | K | R | K | R | R |
| V09 | 1 | 1 | 1 | 0 | 0 | 0 | N | N | N | N | N | N | 1 | 1 | 1 | 0 | 0 | 0 | R | R | R | N | N | N |
| V10 | 0 | 1 | 0 | 0 | 0 | 1 | N | N | N | N | N | N | 1 | 1 | 1 | 0 | 1 | 0 | R | R | K | R | R | K |
| V11 | 0 | 1 | 1 | 0 | 0 | 0 | N | N | N | N | N | N | 1 | 1 | 1 | 1 | 1 | 1 | R | K | R | N | R | N |
| V12 | 0 | 1 | 0 | 1 | 1 | 0 | N | N | N | N | N | N | 0 | 1 | 1 | 1 | 1 | 1 | R | R | R | R | R | R |
| V13 | 0 | 1 | 1 | 1 | 0 | 0 | N | N | N | N | N | N | 1 | 1 | 1 | 1 | 1 | 0 | R | R | R | N | N | N |
| V14 | 0 | 1 | 0 | 1 | 1 | 1 | N | N | N | N | N | N | 1 | 1 | 1 | 1 | 1 | 1 | R | K | R | R | K | R |
| Immersive VR group | | | | | | | | | | | | | | | | | | | | | | | | |
| Subject  No | Pre-VR (multiple-choice questions) | | | | | | Pre-VR (remember[R] / know [K] / none [N]) | | | | | | Post-VR (multiple-choice questions) | | | | | | Post-VR (remember[R] / know [K] / none [N]) | | | | | |
|  | Q1 | Q2 | Q3 | Q4 | Q5 | Q6 | RKN1 | RKN2 | RKN3 | RKN4 | RKN5 | RKN6 | Q1 | Q2 | Q3 | Q4 | Q5 | Q6 | RKN1 | RKN2 | RKN3 | RKN4 | RKN5 | RKN6 |
| I06 | 0 | 1 | 0 | 0 | 1 | 0 | N | N | N | N | N | N | 1 | 1 | 1 | 0 | 1 | 0 | R | R | R | N | R | R |
| I07 | 0 | 1 | 0 | 0 | 0 | 0 | N | N | N | N | N | N | 0 | 1 | 1 | 0 | 1 | 1 | N | R | R | N | R | R |
| I08 | 0 | 1 | 0 | 0 | 1 | 0 | N | N | N | N | N | N | 1 | 1 | 1 | 0 | 1 | 1 | R | R | R | N | R | N |
| I09 | 0 | 1 | 0 | 0 | 1 | 0 | N | N | N | N | N | N | 1 | 1 | 1 | 0 | 1 | 0 | R | R | R | N | R | R |
| I10 | 0 | 1 | 1 | 0 | 0 | 0 | N | N | N | N | N | N | 1 | 1 | 1 | 1 | 1 | 1 | R | R | R | R | R | R |
| I11 | 1 | 0 | 1 | 1 | 0 | 0 | N | N | N | N | N | N | 0 | 1 | 1 | 0 | 1 | 1 | N | R | R | R | R | R |
| I12 | 0 | 0 | 0 | 0 | 0 | 1 | N | N | N | N | N | N | 1 | 1 | 1 | 1 | 1 | 1 | R | R | N | R | R | R |
| I13 | 0 | 0 | 1 | 1 | 0 | 0 | N | N | N | N | N | N | 1 | 1 | 1 | 1 | 1 | 1 | R | K | R | K | R | K |
| I14 | 0 | 0 | 0 | 0 | 0 | 0 | N | N | N | N | N | N | 1 | 1 | 1 | 1 | 1 | 1 | R | R | R | R | R | R |
| I15 | 0 | 0 | 1 | 1 | 0 | 0 | N | N | N | N | N | N | 1 | 1 | 1 | 1 | 1 | 1 | R | R | R | R | R | R |

Six questions were asked, each with four answer choices. *: correct answer. average score, with correct answers of 1 and incorrect answers of 0. Number of responses for each category (remember [R] / know [K] / none [N]).
